# Effect of IMU location on estimation of vertical ground reaction force during jumping

**DOI:** 10.3389/fbioe.2023.1112866

**Published:** 2023-03-20

**Authors:** Jordan A. Kerns, Andrew S. Zwart, Pietro S. Perez, Reed D. Gurchiek, Jeffrey M. McBride

**Affiliations:** Neuromuscular and Biomechanics Laboratory, Department of Public Health and Exercise Science, Appalachian State University, Boone, NC, United States

**Keywords:** center of mass, acceleration, sacrum, velocity, power

## Abstract

**Introduction:** Several investigations have examined utilizing inertial measurement units (IMU) to estimate ground reaction force (GRF) during exercise. The purpose of this investigation was to determine the effect of inertial measurement units location on the estimation of ground reaction force during vertical jumping.

**Methods:** Eight male subjects completed a series of ten countermovement jumps on a force plate (FP). The subjects had an inertial measurement units attached to the sacrum, back and chest. Ground reaction force was estimated from data from the individual inertial measurement units and by using a two-segment model and combined sensor approach.

**Results:** The peak ground reaction force values for the sacrum, back, chest and combined inertial measurement units were 1,792 ± 278 N, 1,850 ± 341 N, 2,054 ± 346 N and 1,812 ± 323 N, respectively. The sacral inertial measurement units achieved the smallest differences for ground reaction force estimates providing a root mean square error (RMSE) between 88 N and 360 N. The inertial measurement units on the sacrum also showed significant correlations in peak ground reaction force (*p* < 0.001) and average ground reaction force (*p* < 0.001) using the Bland-Altman 95% Limits of Agreement (LOA) when in comparison to the force plate.

**Discussion:** Based on assessment of bias, Limits of Agreement, and RMSE, the inertial measurement units located on the sacrum appears to be the best placement to estimate both peak and average ground reaction force during jumping.

## Introduction

The use of inertial measurement units (IMU) for analysis and evaluation of physical activity is a topic that has had extensive research completed and researchers still have many more questions regarding their use. Previous investigators have demonstrated that IMUs provide valid and reliable movement data in tasks including jumping, running, and change of direction (Bergamini et al., 2012; Bailey and Harle, 2014; McGinnis et al., 2016; Schmidt et al., 2016; Gurchiek et al., 2017; MacDonald et al., 2017; Havens et al., 2018; Rantalainen et al., 2019; Toft Nielsen et al., 2019; Zago et al., 2019; Cabarkapa et al., 2022). IMUs have also been utilized to evaluate a multitude of other sports activities in which a force plate (FP) is not viable such as baseball pitching, hitting, alpine skiing, cricket bowling, wheelchair racing and figure skating (Sato et al., 2009; McGinnis and Perkins, 2012; Mapelli et al., 2014; Yu et al., 2016; Fasel et al., 2017; Brice et al., 2018; Boddy et al., 2019; Lapinski et al., 2019; Roell et al., 2019). The defining aspect of the biomechanical analysis of movement is the identification or estimation of a person’s center of mass (COM) acceleration. The use of various procedural approaches that use motion capture and FPs to measure COM acceleration have provided important insight (Gard et al., 2004; Gutierrez-Farewik et al., 2006; Mapelli et al., 2014; Pavei et al., 2017). Acceleration of the COM is known as a product of the resultant ground reaction force (GRF) making FPs an important measurement method, however, there must be another option when a FP is not available. IMUs placed on the body can also be used to estimate COM acceleration but the mounting of the IMU on particular locations of the body would influence whether the IMU acceleration values were valid in terms of the body’s COM acceleration. A limited number of studies have examined how the location of an IMU on the torso influences the validity of estimating ground reaction force in various types of physical activity. Thus, this investigation examined IMU placement on various locations in the vertical jump.

There are several investigations that have calculated various performance variables relating to sprinting utilizing IMUs (Bergamini et al., 2012; Schmidt et al., 2016; Gurchiek et al., 2017; de Ruiter and van Dieen, 2019). This includes estimates of stride length, vertical and horizontal displacement and GRFs. Accelerative running tasks have also been evaluated using IMUs, and in comparison to FP measurements, observable correlations (r = 0.53—0.95; *p* ≤ 0.05) between FP and IMU derived force data were found that lead to the suggestion that IMUs are acceptable devices for measuring these various tasks at low to moderate accelerations (Gurchiek et al., 2017; Zago et al., 2019). Various dependent variables such as force and acceleration have been calculated from IMUs during vertical jumping tasks (McGinnis et al., 2016; MacDonald et al., 2017; Al-Amri et al., 2018; Rantalainen et al., 2019; Toft Nielsen et al., 2019). Jump heights, velocities, and flight time have all been calculated. Aside from kinematic variables measured during relatively simple tasks such as jumping, highly mobile sports, such as alpine skiing, baseball, and discus have all been monitored using IMUs to gather information of the body’s natural movements during sports and provide more useful biomechanical data (McGinnis and Perkins, 2012; Yu et al., 2016; Fasel et al., 2017; Brice et al., 2018; Boddy et al., 2019; Lapinski et al., 2019).

Two variables are needed to estimate GRF based on Newton’s Second Law; mass and acceleration. While mass is a simple measurement to obtain, acceleration provides a challenge. GRF from FP data is considered to be the best option when it comes to calculating COM acceleration (Gutierrez-Farewik et al., 2006). Motion capture is another common method used to estimate GRFs. Estimating COM acceleration *via* motion capture typically involves using a set of reflective markers along the body to estimate the movement of body segments. This estimation allows for a calculation of the COM when combining the mass and movement of each segment into one full body model. Initially, dozens of markers were required, making it restrictive on the types of movements that could effectively be performed. In recent years, technological advancements have led to the use of a single marker placed on the sacrum to calculate the COM using a constant vector offset (Mapelli et al., 2014; Tisserand et al., 2016). By estimating the position of the COM during a movement, it may be possible to capture accurate data using a single IMU. However, given the difficulty and complexity of tracking the COM using an IMU, the placement of IMUs must accurately approximate the COM throughout the entire movement. By placing IMUs on different landmarks across the body, it becomes possible to test their accuracy and compare the data to that obtained from a FP. IMUs could potentially limit the need for the use of FPs when providing data regarding GRFs and force-time curves. IMUs are cheaper, smaller, and easier to transport, making them more accessible than FPs. Given acceleration values it is easy to calculate GRFs from the data given Newton’s second law of motion. The use of IMUs would also allow for the determination of GRFs in situations where FPs are not appropriate.

When comparing IMU data to data collected from a FP, it is imperative to adjust the axes of the IMU to line up with the axes from the FPs (Gurchiek et al., 2017). As the IMU moves and rotates as a result of changes in body position, the axes also change with respect to what the IMUs fixed axes are in the x, y, and z-directions. Therefore, the IMU has its own local coordinate system, while the FP remains on a global coordinate system. In order to align the two sets of axes, the IMU axes must be rotated two times to account for changes in both the vertical and horizontal directions (Gurchiek et al., 2017). Previous researchers compared the data from multiple IMU locations to data collected from FPs in cricket bowling and reported small correlations (Callaghan et al., 2018). The small correlations may have resulted from the lack of axis correction during their analysis (Callaghan et al., 2018). IMU data from a simple walking task was also compared to data collected by a FP (Shahabpoor et al., 2018). By using quaternions to adjust the axes from the data collected using the IMUs, these authors reported that IMUs can effectively measure tri-axial acceleration during walking through a one, two, or three IMU system. The use of quaternions for the evaluation 3D angular kinematics is a means of simplifying the mathematical process during analysis. Quaternions have been described as an efficient algebraic approach compared to others used for angle analysis by avoiding the use of trigonometry and other complex evaluations (Challis, 2020). A previous study used IMUs to measure vertical, horizontal, lateral and resultant force during sprinting and change of direction tasks (Gurchiek et al., 2017). In order to compare the IMU data to the FP data, quaternions were used, and a detailed explanation of the correction process was described and the method was successful.

The purpose of the present study was to determine whether or not an IMU placed on three different positions, thought to be near the centre of mass, could produce valid GRF data in vertical jumping. Data collected from the IMUs were used to compare estimated GRF to FP measurements during vertical jumping. We hypothesised that the sacral placement would be the most accurate for estimating GRF.

## Materials and methods

### The experimental approach to the problem

The subjects wore three commercially available IMU sensors around the estimated COM (BioStampRC; model BRCS01, MC10 Inc., Cambridge, MA, USA). Each IMU had dimensions of 6.6 × 3.4 × 0.3 cm and a mass of 6 g and comprised of three-axis accelerometer (range: ± 16 g), three-axis gyroscope (range: ± 2,000o/s), and analog front end for sEMG and ECG measurement (not applicable for this study). The IMUs were set to a sampling rate of 250 Hz and data were written in an onboard non-volatile memory, which could later be uploaded to the BioStampRC online portal *via* Bluetooth^®^ Smart Connectivity and downloaded to a computer for analysis. The IMUs were managed through the Investigator App (v1.4.7. released 9 April 2018) installed on an Android tablet provided by BioStampRC system. Data obtained from the FP (AMTI, Watertown, MA, sampling rate: 1,000 Hz) was collected simultaneously for direct GRF measurements for comparison to IMU sensor estimated GRF.

### Subjects

Eight recreational active men (age: 25.75 ± 3.06; height: 182.19 ± 7.34 cm; mass: 83.03 ± 13.13 kg) volunteered to participate in this study. Subjects included in the study were between the ages of 18 and 35 years old, healthy, and reported no musculoskeletal injury within the past 6 months prior to testing. All subjects provided written consent to participate in the study which was approved by the Appalachian State University Institutional Review Board.

### Procedures

The subjects visited the laboratory on one occasion. Age, height and weight were obtained. The IMUs were placed on the sacrum (L4-L5), upper back (on the spine as a position in line with the xiphoid process) and the chest (xiphoid process) of each participant using double-sided adhesives. The locations were used together to limit errors in COM estimation based upon previous research suggesting these placements may have certain limitations when used individually when compared to the use of them together since COM varies constantly during limb movement (Gullstrand et al., 2009; Cerrito et al., 2015; Cain et al., 2016; Gurchiek et al., 2017). Subjects first performed three practice vertical jumps. Subjects were then instructed to perform ten maximal effort vertical countermovement jumps with their hands placed on their hips in order to avoid any disturbance with IMU measurements because of arm movement. Subjects stood on a FP and performed each jump after an acoustic signal with approximately 15 s between each jump. The subjects were told to stay as still as possible (standing stationary) between jumps so that the data estimates could represent a constant force of the participant standing.

### Data processing

Custom scripts written in MATLAB (MathWorks, Natick, MA) were used to perform data analysis. The FP data was re-sampled to 250 Hz using the analysis scripts so that it would be comparable to IMU estimates. IMU data were low-pass filtered at 15 Hz to remove any bias and time-synchronized with FP data *via* cross-correlation (McGinnis et al., 2015). Using IMU data, the GRF was then estimated as previously described (Gurchiek et al., 2017). Accelerometer data during the previously identified still intervals before each jump, provide an estimate of initial sensor attitude. The initial estimate is then used to estimate the subsequent sensor attitude *via* strapdown integration of angular rate found from the gyroscope. Instantaneous sensor attitude allowed for expression of sensor referenced acceleration in the world frame to appropriately compare the vertical component of GRF between IMU and FP estimates. GRF was estimated by scaling IMU global reference frame acceleration by the participant’s mass. This approach assumed the sensor acceleration sufficiently resembled the COM acceleration. A two-segment model and combined sensor approach was also considered where the average of the back and chest acceleration modelled the upper body acceleration and the sacral sensor modelled the lower body (Eq. 1). GRF was then estimated by the sum of the upper body and lower body acceleration scaled by estimates of their respective mass (de Leva, 1996).

$${GRF}_{combined}=\frac{k}{2}\left(GRF_{chest}+GRF_{back}\right)+\left(M-k\right){GRF}_{sacrum}$$ (1)

In Equation 1, k is the constant equal to the subjects’ body mass (M) times 0.4911, as described previously (de Leva, 1996). Jump start and take-off instants were determined independently for each IMU. The take-off instant was defined for the FP as the instant GRF went below 10 N. Relative segment movement prevents a similar threshold-based identification of take-off for the sensors. Instead a physics-based approach was used and defined the take-off instant for sensor estimates of GRF as the instant of peak velocity. To identify the jump start, the instant of minimum GRF between the still interval and the peak GRF before take-off was found. Working backwards in time from this instant, jump start was defined as the first instant GRF exceeded two standard deviations below the participant’s GRF while standing still. Peak GRF and average GRF were determined for each method using the respective start and take-off instants. GRF estimates for each method were resampled to allow ensemble averaging across subjects for visual comparison of instantaneous GRF.

### Statistical analysis

All variables are reported as the mean ± standard deviation. Two-tailed t-tests were run to find each participant’s average peak and total mean forces over all ten trials for each sensor, indicating which sensors were significantly different from the FP. Statistical significance for all statistical tests was set to a level of ≤0.05. Correlations between the FP GRF recordings and the IMUs were also calculated. The absolute error in the IMU estimate was qualified using RMSE and relative error (absolute percent error). To determine how well IMU estimates scaled with that of the reference FP, Pearson product moment correlation coefficient (r) was calculated. Bland-Altman 95% Limits of agreement (LOA) were used to assess the reliability of the IMU methods. For the Bland-Altman analysis, the normality of the difference distributions was tested using the Shapiro-Wilk test. The error in the IMU estimate of peak and average GRF from RMSE and r across all subjects was used to assess the ability of the IMU methods to estimate GRF. Differences throughout the force-time curves between the IMUs and the FP were assessed through a SPM (statistical parametric mapping) 2-way (condition × time) repeated measures analysis of variance (ANOVA) with α = 0.05. An ANOVA post-hoc analysis using the SPM *t*-test and Bonferroni correction was then utilized to determine the location of any differences along the force-time curves. The assumptions for linear statistics were met, and statistical significance for all analyses was defined by *p* ≤ 0.05.

## Results

### Peak GRF

**FIGURE 1:**
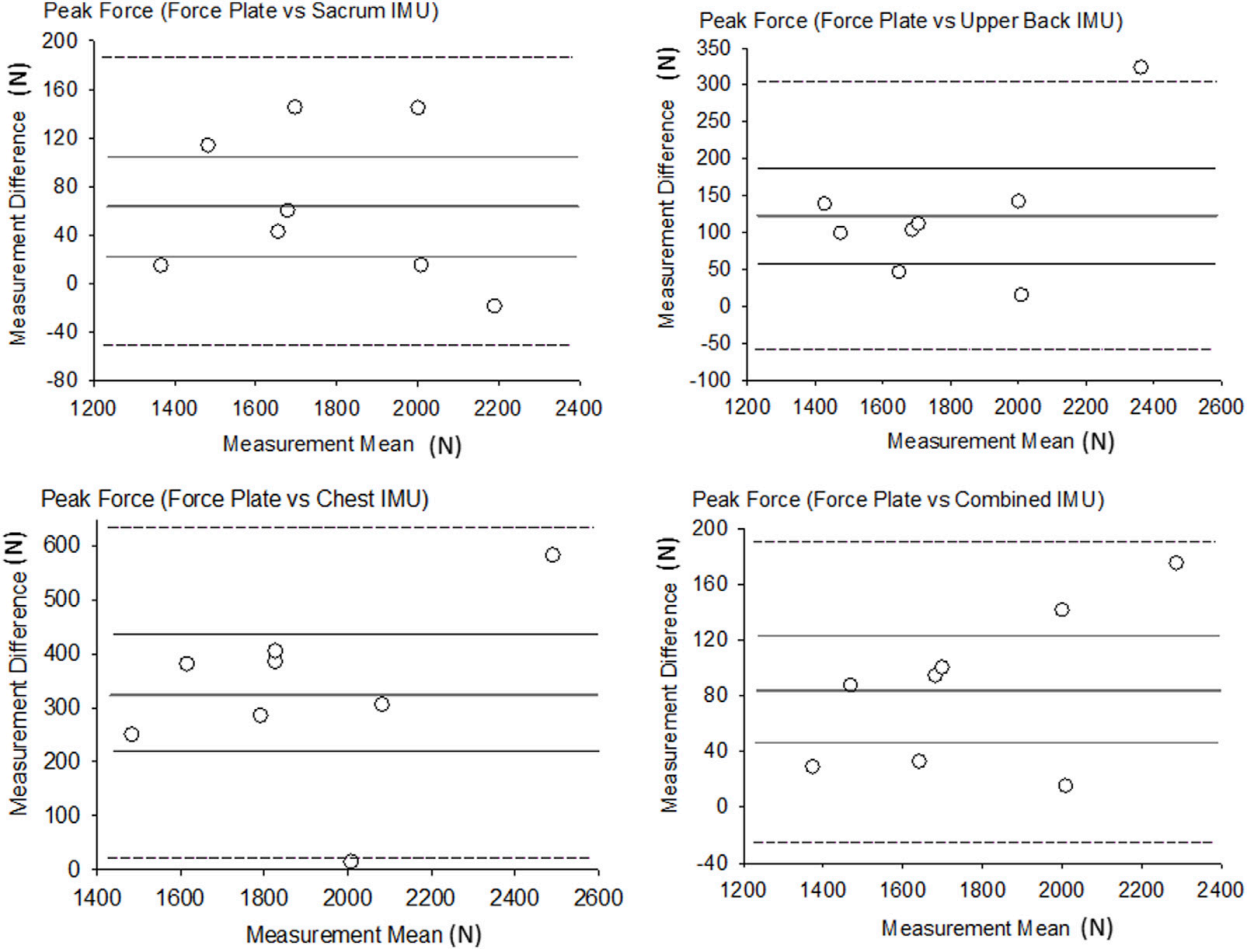
Bland-Altman plots of peak force. Thicker dark line = Bias, Thinner gray lines = Bias 95% CI, Dotted lines = LOA.

The range of the peak GRF from each sensor was between 1,792 ± 278 N to 2,054 ± 346 N and was compared to the FP measurement of 1,727 ± 291 N (Table 1 near here). The *R* ^2^ values for the sacrum, upper back, chest and combined were 0.96, 0.94, 0.79 and 0.98 respectively. The range of the bias between the sensors and FP was between 57 N and 162 N and the relative error was the lowest for the sacrum location (3.9%) and highest for the chest location (15.9%). All IMU estimates of peak force from the sacrum, upper back, chest, and combined showed significant relationships (*p* ≤ 0.003, r = 0.89–0.99). The RMSE for peak force was between 88 N and 360 N, which indicates that the sacrum achieved the smallest differences for GRF estimates, while the chest IMU was the least accurate in estimating GRF. Figure 1 shows the results of the Bland-Altman analysis, where all measurement differences were normally distributed and showed no linear trend with the measurement means (Figure 1 near here).

**TABLE 1:** Inertial Measurement Unit (IMU) estimated peak force (PF) during a vertical jump in comparison to the force plate (FP) value of 1,727 ± 291 N (mean ± sd). Bias,Bland-Altman 95% limits of agreement (LOA), root mean square error of IMU estimate (RMSE), relative error (RE) and Pearson product moment correlation between IMU and FP measure.

Location	IMU PF (N)	Bias (N)	LOA (N)	RMSE (N)	RE (%)	r (*p*-value)
Sacrum	1,792 ± 278	70	−44,184	88	3.9	0.98 (<0.001)*
Upper Back	1,850 ± 341	92	−62, 307	150	6.6	0.97 (<0.001)*
Chest	2,054 ± 346	162	3, 650	360	15.9	0.89 (0.003)*
Combined	1,812 ± 323	57	−28,198	100	4.7	0.99 (<0.001)*

### Average GRF

**FIGURE 2:**
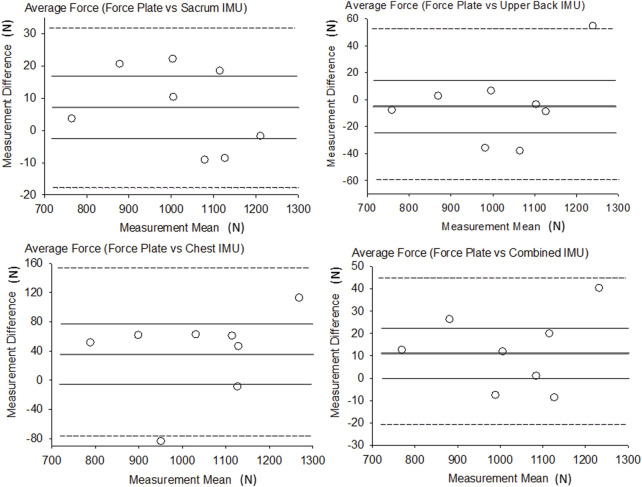
Bland-Altman plots of average force. Thicker dark line = Bias, Thinner gray lines = Bias 95% CI, Dotted lines = LOA.

IMU estimates of average force ranged from 1,015 ± 158 N to 1,057 ± 165 N and relative error ranged from 1.2% (sacrum) to 5.8% (chest) (Table 2 near hear). The *R* ^2^ values for the sacrum, upper back, chest and combined were 1.0, 0.96, 0.86 and 0.98 respectively. The RMSE for average force was the highest in the chest IMU (67 N) and the lowest in the sacrum IMU (14 N). Each IMU location as well as the combined IMUs demonstrated significant relationships (*p* ≤ 0.001, r = 0.93–1.00). Figure 2 shows the results of the Bland-Altman analysis for average GRF along with the LOA and plot of each participant compared to the average bias, and all measurement differences were normally distributed, and no linear trend was shown (Figure 2 near here). The bias in the IMU estimate of average force was between 8 N and 30 N.

**TABLE 2:** Inertial Measurement Unit (IMU) estimated average force (AF) during a vertical jump in comparison to the force plate (FP) value of 1,019 ± 147 N (mean ± sd). Bias,Bland-Altman 95% limits of agreement (LOA), root mean square error of IMU estimate (RMSE), relative error (RE) and Pearson product moment correlation between IMU and FP measure.

Location	IMU AF (N)	Bias (N)	LOA (N)	RMSE (N)	RE (%)	r (*p*-value)
Sacrum	1,026 ± 142	8	−4, 27	14	1.2	1.00 (<0.001)*
Upper Back	1,015 ± 158	20	−20,60	27	1.9	0.98 (<0.001)*
Chest	1,057 ± 165	30	1,121	67	5.8	0.93 (0.001)*
Combined	1,031 ± 149	12	−9, 41	20	1.6	0.99 (<0.001)*

### Ensemble force-time curves

**FIGURE 3:**
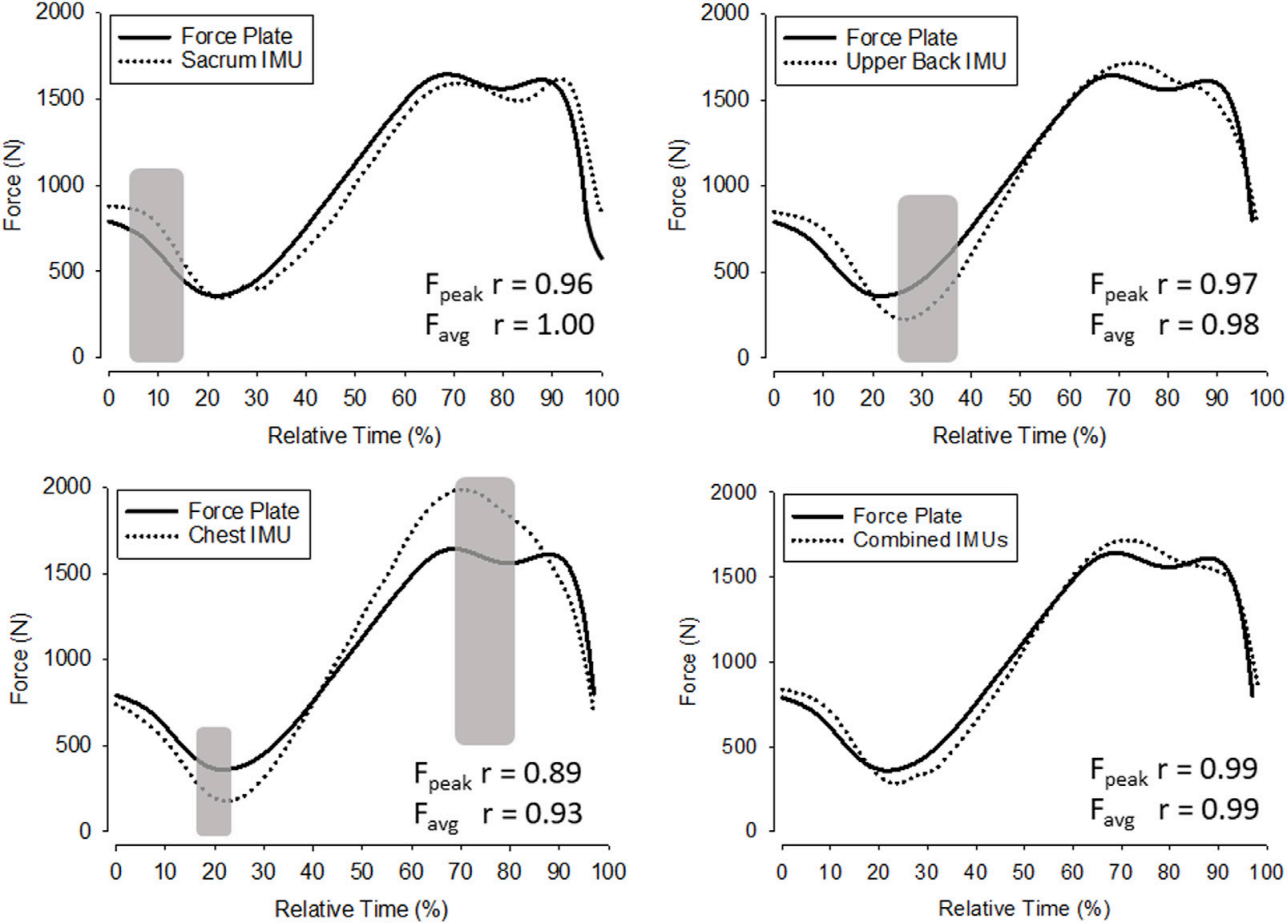
Ensemble average force-time curves. Gray shaded areas indicate significant differences between the respective FP force-time curve values and the IMU force-time curve values (*p* ≤ 0.05).

The GRF estimates for each participant were down-sampled to 100 Hz in order to reduce noise in the force-time curve, and then averaged together for each sensor (Figure 3 near here). The ensemble curves of the IMUs illustrate the points in time where the sensors deviated from FP estimates. Upper back and chest achieved comparatively lower correlations values with the FP (r = 0.98 and 0.93 for average GRF), while the combined system of all three IMUs achieved the most consistent and significant (*p* < 0.001) estimates of GRF throughout the jump. At the beginning of the jump, upper back and chest IMUs tended to underestimate GRF, while sacrum sensor tended to overestimate (*p* > 0.05 at 10% of Relative Time), yet towards the end of the jump phase the chest IMU greatly overestimated force production (*p* > 0.05 at 70%–80% of Relative Time).

## Discussion

The purpose of this study was to determine whether or not an IMU placed near the centre of mass, in three surrounding locations, could produce valid GRF data in vertical jumping. The results demonstrate that the location of an IMU significantly alters estimates of GRF. An IMU placed on the sacrum provided the best estimates of GRF in comparison to FP data. As in previous studies, a sacral mounted IMU was successfully used to calculate peak GRF, average GRF, and ensemble force-time curves during jumping in the present study (Cerrito et al., 2015; Tisserand et al., 2016). While the sacral IMU provided the most accurate data, this is the first study to suggest that sacral, upper back, and chest IMU measurements could individually provide viable alternatives to a FP for estimating GRF during jumping. The sacral IMU shows statistical correlational significance in peak GRF (*p* < 0.001) and average GRF (*p* < 0.001) and acceptable error examining the Bland-Altman 95% Limits of agreement (LOA) when compared to the FP. The upper back IMU also shows statistical correlational significance in peak GRF (*p* < 0.001) and average GRF (*p* < 0.001). Of the three placements, the sacral IMU proved the most accurate with the smallest error in both the peak (r = 0.98) and average GRF (r = 1.00). When the sacral, upper back, and chest measures were combined, they produced an ensemble force-time curve nearly identical to that of the FP (*p* < 0.001). Variables directly calculated from GRF such as impulse and jump height would have the same results in LOA which may be of particular interest to practitioners in the field. However, it should be noted that specific instances of appropriate usage of an IMU and it is location on the body must ultimately be decided by the clinician or practitioner. Appropriate determination of clinical or physical performance relevance utilizing this device will vary as different circumstances of comparison need differing levels of precision.

The necessity for orientation correction of IMU data is further confirmed by this study. Following the protocol set forth in previous studies regarding IMUs, quaternions were effectively used to line up the IMU and FP data in this study (Gurchiek et al., 2017). After recording static measures of the IMU placed on the sacrum, upper back, or chest while standing in the upright at rest position, the IMU can be effectively “zeroed” to match the FP. By aligning two of the axes from the IMU data during the static trial with the same two axes from the FP using two simple rotations, the x, y, and z directions matchup between the two sets of data. Throughout the vertical jumping task, the IMU data is time sequenced to the FP data which allows for a consistent axis throughout the movement. As a result, the calculation of GRFs becomes possible as the IMU has a frame of reference that matches up with the plane of movement, in this case the floor. The significant findings of the current study confirm that correct orientation of the device is critical for accurate analysis of IMU data.

With a single IMU providing accurate calculations of centre of mass during vertical jumping, the multiple marker motion capture system becomes redundant. Upwards of thirty markers were frequently used to calculate the centre of mass using a motion capture system (Tisserand et al., 2016). While that still may be a viable system for researchers looking to track the change of the centre of mass over time or an array of other kinematic variables, a single IMU placed on the sacrum may provide accurate results. A single IMU can be used for the purpose of calculating GRFs or other many other variables, such as acceleration, that are used to calculate secondary variables during a vertical jumping task. IMUs are cheaper, smaller, and easier to transport, than systems using multiple markers or FPs, making them accessible to a larger population. In situations where FPs may exceed a budget, IMUs are a cheaper option that can be used instead to provide accurate data regarding GRFs and force-time curves. Tasks that occur over a large area and are not confined to any one place could benefit from the mobility of IMUs, such as tracking a football match or sprint performance. GRFs are understood to be essential in high-level sprinting performance, but the mechanisms with which sprinters generate that force is relatively unknown. Most world-class sprinters take 5–7 s to reach their top speed. As a result, FPs are insufficient in tracking GRFs throughout the entire acceleration phase (Nagahara et al., 2014). Using IMUs as a substitute for FPs would allow GRFs to be tracked throughout the acceleration phase and would allow GRFs in vertical jumping to be tested outside of the laboratory setting, expanding upon current research (Rabita et al., 2015; Cabarkapa et al., 2022).

The mass variance in centre of mass during locomotion remains the single biggest limitation in producing valid data. Human COM location within the body is variable from participant to participant and from movement to movement. Including more subjects in the current study could account for the wide variance and more testing of IMUs may help researchers better understand the location of COM during various activities. It is worth noting that centre of gravity is synonymous with centre of mass when examining human subjects in a uniform gravitational field. Early research on the centre of gravity in sit to stand tasks shows the variability of the centre of gravity in simple everyday tasks (Murray et al., 1967). When comparing the centre of mass between a walking and a running task, the centre of mass has been shown to change by as much as 0.073 m (Lee and Farley, 1998). When examining a high jump or pole vault task, the centre of mass is located outside of the body. While the body passes over the top of the bar, the centre of mass passes below the bar due to the inverted U-shape assumed during the task which results in a displaced centre of mass (Ae et al., 2008). For this reason, the implications of this study may be limited without further research.

There are additional limitations with the use of IMUs. Consistency of IMU placement remains a significant challenge to IMU data. FPs remain consistent between subjects while IMUs are placed in varying positions on every participant. This study is further limited by the small sample size of exclusively taller and fit men. In other populations, soft tissue movement may cause varied acceleration values. Future research should investigate the efficacy of using the sacral IMU placement for calculation of GRFs in sprinting and other sport specific tasks as well as the efficacy of IMU tracking on other populations.

## Conclusion

In situations where a three IMU system may not be possible, the sacral location shows the most promising results during the countermovement jumping task completed by a small group of men. Despite the rapid change in centre of mass during complex movements, the data presented suggests that it remains close to the sacrum, and not the upper back or chest. Based on trunk flexion and extension during a hopping task, this data is consistent with our hypothesis. During a counter movement jump, the trunk begins in a neutral position, flexes forward to around 40°, and promptly returns to neutral before take-off (Lees et al., 2004). This rapid change in position creates additional acceleration measured in the upper back and chest IMUs which are distal to the axis of rotation when compared to the proximal placement of the sacral IMU. Upper back and chest IMUs may be more effective in a running or gait task in which the trunk remains in a more neutral and upright position throughout the duration of the movement while the sacrum proves to be the best placement when evaluating movements with varying chest movement, including vertical jumping. This data provides important information pertaining to the desired location of an IMU for collection of valid data pertaining to training athletes or perhaps in a rehabilitation setting. Perhaps previous publications should reevaluate their data in light of the current investigations findings based on their specific IMU placement locations.

## Data Availability

The raw data supporting the conclusion of this article will be made available by the authors, without undue reservation.
